# Polysaccharide Composites with *Rosa canina* for Sustained Anti-Inflammatory Skin Therapy

**DOI:** 10.3390/polym17121707

**Published:** 2025-06-19

**Authors:** Narcis Anghel, Irina Apostol, Ioana Plaesu, Alice Mija, Natalia Simionescu, Adina Coroaba, Iuliana Spiridon

**Affiliations:** 1 “Petru Poni” Institute of Macromolecular Chemistry, Grigore Ghica-Voda No. 41A, 700487 Iasi, Romania; apostol.irina@icmpp.ro (I.A.); adina.coroaba@icmpp.ro (A.C.);; 2 Faculty of Chemistry, “Alexandru I. Cuza” University, Bvd. Carol I No. 11, 700506 Iasi, Romania; 3 Institut de Chimie de Nice, Université Côte d’Azur, UMR 7272 CNRS, 28 Av Valrose, 06108 Nice, France

**Keywords:** guar gum, dextran, lignin aspartate, *Rosa canina* extract, drug release, skin applications

## Abstract

This study presents novel skin-compatible biomaterials based on guar gum and dextran sulfate matrices, incorporating softwood lignin, lignin esterified with aspartic acid, and *Rosa canina* extract. The materials were prepared via casting and evaluated for physicochemical, mechanical, and biological properties. Spectroscopic analyses confirmed successful lignin esterification, with new carbonyl and amide peaks and a nitrogen signal (3.83%) detected. *Rosa canina* extract enhanced the Young’s modulus from 1.42 MPa to 3.18 MPa and reduced elongation at break from 34.88 mm to 25.19 mm. The combination of esterified lignin and *Rosa canina* showed the greatest mechanical reinforcement (3.74 MPa modulus, 23.78 mm elongation). Swelling capacity decreased from 0.40 to 0.23 g water/g material and followed pseudo-second-order kinetics (R^2^ = 0.991–0.998). The release of *Rosa canina* bioactives followed the Makoid–Banakar model, indicating a transition from rapid to sustained release. All formulations exhibited anti-inflammatory activity with over 45% protein denaturation inhibition, peaking at 61.58% for the *Rosa canina*-only sample. In vitro biocompatibility assays demonstrated over 80% cell viability, confirming the potential of these biomaterials for dermal applications.

## 1. Introduction

The human skin, the body’s largest organ, serves as a protective barrier, preventing water loss and shielding against environmental factors such as solar UV radiation and airborne nanoparticles. These external aggressors contribute to increased skin stiffness, dryness, and premature aging, ultimately affecting both its appearance and physiological functions. The delicate balance of hydration and protection is vital for maintaining healthy skin, making effective skincare practices essential not only for esthetics but also for overall well-being [1].

As living standards improve, the demand for advanced cosmetic products, particularly facial masks, has increased. Traditional wet facial masks, although widely used for their hydrating and nourishing properties, pose concerns due to their composition containing preservatives and antimicrobial agents and their non-biodegradable packaging. Additives such as methylparaben and benzalkonium chloride are included in facial masks to prevent the growth of bacteria, fungi, and other microorganisms that could compromise product safety. Methylparaben serves as an effective preservative, especially in water-based formulas and those containing natural ingredients, helping to extend shelf life. Benzalkonium chloride plays a dual role as both an antimicrobial and disinfectant, supporting product hygiene—particularly in reusable or jar-packaged formulations. While these compounds provide stability and protection, they can also trigger skin irritation or allergic reactions in sensitive individuals, which is why they are used in very low, strictly regulated concentrations. Furthermore, their environmental impact is becoming increasingly problematic. In response to these challenges, dry facial masks have gained popularity as a sustainable alternative. By eliminating preservatives and incorporating biodegradable packaging, dry masks address both health and environmental concerns. However, the high cost of production and the degradation of active ingredients during processing present obstacles to their widespread adoption, highlighting the need for improved and cost-effective manufacturing techniques [2].

With sustainability becoming a key driver in the cosmetic industry, research has focused on natural and biodegradable compounds [1]. Among the various biopolymer-based materials explored, polysaccharide systems have demonstrated great potential due to their biocompatibility, versatility, and multifunctionality [3]. Guar gum and dextran sulfate, in particular, have emerged as promising candidates for cosmetic applications due to their natural origin and their ability to enhance the mechanical and functional properties of skincare materials. These polysaccharides not only serve as excellent film-forming agents but also contribute to the elasticity and swelling behavior of the final material, allowing it to adapt to the skin’s morphology. This ensures enhanced hydration retention and increased comfort during application. Guar gum, a galactomannan polysaccharide, is widely recognized for its high viscosity, moisture-retaining ability, and ability to form stable hydrogels. It also exhibits good compatibility with other biopolymers, improving the durability and performance of composite skincare materials [4]. Similarly, dextran sulfate, a sulfated polysaccharide with strong anionic properties, enhances material stability, binding capacity, and biocompatibility, making it well suited for the controlled delivery of active ingredients [5].

Despite their numerous advantages, polysaccharide-based materials such as guar gum and dextran sulfate exhibit several limitations that must be addressed to fully achieve their potential in advanced cosmetic formulations. One major drawback is their inherently poor mechanical strength, which can compromise the durability and structural integrity of the final product under physical or environmental stress. Additionally, these biopolymers are often sensitive to variations in humidity, pH, and temperature, leading to changes in viscosity, swelling behavior, or degradation rate over time. Their natural origin also introduces batch-to-batch variability, which can affect the consistency and reproducibility of cosmetic formulations. Furthermore, while their biodegradability is advantageous from a sustainability perspective, uncontrolled or rapid degradation may shorten shelf life or hinder the sustained release of active ingredients. Polysaccharide films also tend to exhibit low moisture and gas barrier properties, limiting their protective capacity in skincare applications. Lastly, the limited diversity of functional groups in native polysaccharides restricts their ability to be tailored for specific performance requirements, often necessitating chemical modification or hybridization with other materials. These challenges underscore the need for further research into the structural optimization and functional enhancement of polysaccharide systems for next-generation cosmetic technologies.

Further developments in polysaccharide-based materials have included the incorporation of lignin and its chemically modified form, lignin aspartate, as fillers to improve structural stability. Lignin, a naturally occurring aromatic polymer found in plant biomass, has gained attention for its ability to enhance mechanical properties and biodegradability in composite materials. Additionally, its UV-blocking capability, pH-sensitive nature, and chemical stability make it valuable in biomedical applications such as drug delivery systems and tissue regeneration [6,7]. However, lignin’s hydrophobic nature limits its interaction with hydrophilic matrices, often leading to material inconsistencies. To address this issue, lignin was modified through esterification, producing lignin aspartate. This modification increases hydrophilicity through amino and carboxyl groups, allowing lignin to interact more effectively with biologically active compounds, such as those found in *Rosa canina* aqueous extract. As a result, the release of active ingredients from the matrix is slow, extending their beneficial effects on the skin. However, this modification also affects polymer distribution within the matrix, which can impact the homogeneity and mechanical properties of the material [7,8,9].

To further enhance the bioactivity of the material, *Rosa canina* aqueous extract was incorporated for its well-documented anti-inflammatory, antioxidant, and skin-regenerating properties. This botanical extract is rich in flavonoids, polyphenols, and vitamin C and plays a crucial role in neutralizing free radicals, reducing inflammation, and promoting collagen synthesis [10,11]. These attributes make it particularly valuable in skincare applications aimed at preserving skin elasticity, reducing signs of aging, and improving overall skin resilience. Additionally, the natural origin of *Rosa canina* aligns with the current shift toward cleaner, eco-friendly cosmetic formulations that prioritize both efficacy and sustainability [12].

The integration of these components aims to create a balanced material that combines biocompatibility, mechanical stability, and the controlled release of active ingredients. The inclusion of dextran sulfate and guar gum ensures that the material remains flexible and maintains its ability to swell upon contact with the skin, thereby enhancing hydration and overall skin comfort. Meanwhile, the interaction between lignin aspartate and *Rosa canina* extract modulates the release kinetics of bioactive compounds, prolonging their therapeutic effects. This study evaluates the structural, mechanical, and functional characteristics of the formulated materials, emphasizing their potential use in cosmetic applications where sustainable and high-performance skincare solutions are increasingly sought after.

The novelty of this work resides in the strategic integration of enzymatically esterified lignin and *Rosa canina* extract into a dual-polysaccharide matrix (guar gum/dextran sulfate), providing a synergistic platform that enhances mechanical strength, modulates bioactive release, and ensures cytocompatibility. This approach directly addresses the current limitations in polysaccharide-based skin biomaterials, such as poor filler dispersion, rapid drug release, and limited anti-inflammatory efficacy.

## 2. Materials and Methods

### 2.1. Materials

Dextran sulfate sodium salt (DexS), guar gum (Gu), methanol, dimethyl sulfoxide (DMSO), aspartic acid, lipase from Candida rugosa (2 U/mg), ethyl ether, aluminum chloride (AlCl_3_), and sodium carbonate (Na_2_CO_3_) were all purchased from Sigma-Aldrich (St. Louis, MO, USA). Folin–Ciocalteu reagent, gallic acid, and quercetin were acquired from Fluka™ (Muskegon, MI, USA), while *Rosa canina* aqueous extract (RC) was obtained in the laboratory by extraction of 10 g dried fruits in 150 mL water (60 °C) for 12 h, followed by evaporation under reduced pressure at 40 °C to obtain 3.7 g of aqueous extract. The softwood lignin (Li) was obtained from Södra Cell, Vaxjo, Sweden, by the regular Lignoboost process.

### 2.2. Lignin Esterification Reaction

Li was esterified with aspartic acid, through an enzyme-catalyzed reaction, according to the method presented in a previous published paper [13]. Lignin (1 g) and aspartic acid (1 g) were introduced in 50 mL DMSO. After complete dissolution, lipase (200 mg) was added, and the reaction components were gently mixed at room temperature for 72 h. To remove the solvent, both solutions were placed in an oven set at 70 °C under a pressure of 40 mbar. Once the solvent had evaporated, the resulting compound was thoroughly washed multiple times with ethyl ether and water. Finally, the lignin ester (LiAs) was dried at 60 °C and ground into a fine powder. The obtainment yield of LiAs was 64%.

### 2.3. Material Obtainment

Solutions of Gu and DexS (concentrations of 0.5% in distilled water) were prepared. These solutions were mixed together until complete homogenization. Then, RC extract, Li, and LiAs were added one by one into the prepared polymeric matrix solution and mixed at room temperature for 1 h to assure their complete dispersity. A 25 mL volume of each mixture was poured into Petri dishes, and the materials were obtained by the casting method. The materials were dried 48 h at room temperature under ambient airflow.

The casting method employed for film formation in this study was selected due to its suitability for polysaccharide-based systems and botanical extracts, such as *Rosa canina*. Solvent casting is widely reported in the literature as an effective technique for obtaining homogeneous biopolymer films with preserved bioactivity [14,15]. Unlike thermal extrusion [16] or spray drying [17], which may degrade thermolabile compounds, casting allows slow solvent evaporation at ambient conditions, thus maintaining the integrity of both the matrix and incorporated actives. Alternative methods, such as electrospinning [18] or knife coating [19], though useful for achieving specific microstructures or thickness control, often require specialized equipment and may not be ideal for polysaccharide blends with high viscosity or complex filler content. Therefore, the simple, scalable, and gentle nature of solvent casting renders it particularly appropriate for developing multifunctional dermal biomaterials.

**Figure 1:**
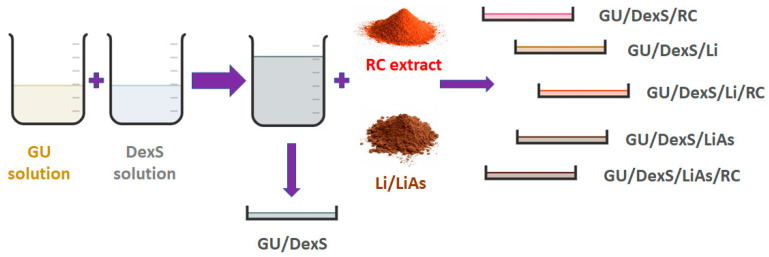
Schematic representation of material obtainment.

The schematic representation of material obtainment is presented in Figure 1, while the mass ratios of the material components are presented in Table 1. The choice of 0.8 mass ratio for fillers was based on prior optimization studies for maintaining film homogeneity and mechanical integrity.

### 2.4. Total Phenolic Content (TPC) of Rosa Canina Extract

The TPC of RC extract was determined using the Folin–Ciocalteu method as it was described by Aryal et al. [20] with slight modifications. Briefly, 0.5 mL of the sample extract solution (concentration of 1 mg/mL) was mixed with 2 mL of Folin–Ciocalteu reagent (1:10 dilution with deionized water). After allowing the mixture to react for 3 min at room temperature, 4 mL of Na_2_CO_3_ solution (7.5% *w*/*v*) was added. The reaction mixture was incubated in the dark at room temperature for 60 min to allow for complete color development. The absorbance of the resulting blue complex was measured at 760 nm using a UV–Vis spectrophotometer (Jenway 6405). A blank was prepared using deionized water in place of the sample extract. A standard calibration curve was generated using gallic acid as the reference compound. Gallic acid solutions of varying concentrations (0.0024–0.156 mg/mL) were prepared in deionized water. Each concentration was treated in the same manner as the samples, including the addition of the Folin–Ciocalteu reagent, Na_2_CO_3_ solution, and incubation period. All measurements were performed in triplicate. The TPC was expressed as mg/g of gallic acid equivalents in milligrams per gram (mg GAE/g) of dry extract. For the obtained extract, the total phenolic content was 0.089 ± 0.0021 mg GAE/g dry RC extract.

### 2.5. Total Flavonoid Content (TFC) of Rosa Canina Extract

The TFC of RC extract was determined using the AlCl_3_ colorimetric method [21] with a few modifications. A 2 mL volume of the sample extract solution (concentration of 1 mg/mL) was mixed with 2 mL of AlCl_3_ solution (2% *w*/*v* in methanol). The mixture was incubated at room temperature for 40 min in the dark to allow for the formation of a stable yellow complex. The absorbance of the solution was measured at 430 nm using the UV-Vis spectrophotometer. A blank solution was prepared using methanol instead of the sample extract. A standard calibration curve was prepared using quercetin as the reference. Quercetin solutions with varying concentrations (0.5–100 mg/L) were prepared in methanol. Each concentration was subjected to the same procedure, including reaction with the AlCl_3_ solution and the same incubation period. All measurements were performed in triplicate. The TFC was expressed as mg/g of quercetin equivalents in milligrams per gram (mg QE/g) of dry extract. For the obtained extract, the total flavonoidic content was 0.071 ± 0.0037 mg QE/g dry RC extract.

### 2.6. ^13^C Nuclear Magnetic Resonance (^13^C NMR)

The solid-state ^13^C NMR spectra were recorded on a Bruker AvanceHD-400 MHz NMR spectrometer operating at a ^13^C resonance frequency of 106 MHz, equipped with a commercial Bruker double-channel probe. ^13^C NMR spectroscopy was performed to investigate the structural differences between unmodified lignin (Li) and lignin esterified with aspartic acid (LiAs). Approximately 10–20 mg of each dried sample was packed into 3.2 mm zirconium dioxide rotors and spun at a Magic Angle Spinning (MAS) rate of 15 kHz to detect chemical shifts indicative of successful esterification. The CP technique was employed with a ramped 1H-pulse, starting at 100% power and decreasing to 50% during the contact time (2.5 ms) to avoid Hartmann–Hahn mismatches. A dipolar decoupling GT8 pulse sequence was applied during the acquisition time to enhance resolution. For an improved signal-to-noise ratio in the ^13^C CPMAS experiment, 30 K scans were accumulated with a 2.5 s delay. The ^13^C chemical shifts were referenced to tetramethylsilane and calibrated using the adamantane –CH signal, set at 38.48 ppm. Special attention was given to the appearance of new signals in the carbonyl region (~170–180 ppm) and aliphatic region (~30–60 ppm), which are associated with the aspartic acid moieties and confirm the formation of ester and/or amide linkages.

### 2.7. X-Ray Photoelectron Spectroscopy (XPS)

X-ray photoelectron spectroscopy was performed to determine the elemental composition and surface chemical states of lignin and its esterified form, particularly the incorporation of nitrogen-containing groups.

XPS was carried out on an Axis Nova device (Kratos Analytical, Manchester, UK), using AlKα radiation with 20 mA current and 15 kV voltage (300 W), and a base pressure of 10^−8^ ÷ 10^−9^ Torr in the sample chamber. The incident monochromated X-ray beam was focused on a 0.7 mm × 0.3 mm area of the surface. The XPS survey spectra for the samples were collected in the range of −5 ÷ 1200 eV with a resolution of 1 eV and a pass energy of 160 eV. The high-resolution spectra for all the elements identified from the survey spectra were collected using a pass energy of 20 eV and a step size of 0.1 eV. The acquisition of all spectra and survey spectra processing were performed using the ESCApe software.

### 2.8. Fourier-Transform Infrared Spectroscopy (FTIR)

FTIR spectroscopy was used to analyze functional groups and intermolecular interactions in the materials, and to verify the successful incorporation of lignin, lignin aspartate, and *Rosa canina* extract.

A Vertex 70 FTIR spectrometer from Brüker, equipped with an ATR (Attenuated Total Reflectance) device (ZnSe crystal), was used to recorder the FTIR spectra of the obtained materials. These spectra were analyzed in the range of 4000–600 cm^−1^, with an average of 64 scans and a spectral resolution of 2 cm^−1^, at a 45 angle of incidence.

### 2.9. Material Density

To evaluate the compactness and structural integration of fillers within the polysaccharide matrix, materials’ densities were calculated.

The density of the obtained materials was determined using the pycnometer method. The volume of the solid material was indirectly measured by assessing the amount of ethanol displaced by it. Its density was then calculated using Equation (1):

(1) $$d_{s}=\frac{m_{s}}{v_{s}}=\frac{m_{s}}{\left(m_{a}+m_{s}-m_{as}\right)\times d_{ethanol}},(g\cdot {cm}^{-3})$$

where *m_s_* represents the weight of the studied material, *v_s_* represents the volume of the studied material, *m_a_* represents the weight of the pycnometer completely filled with ethanol, *m_as_* represents the weight of the pycnometer, which contained the studied materials (the rest free volume of the pycnometer is filled with ethanol), and *d_ethanol_* represents the ethyl alcohol density.

The samples were cut using a punch in order to obtain approximately equal masses. The density of ethanol was 0.789 g/cm^3^. For each material, the density values were obtained by analyzing three test specimens. The results are reported as the average of the three measurements, accompanied by the standard deviation (SD).

### 2.10. Tensile Properties

To assess the mechanical performance of the films, including strength, stiffness, and flexibility, as influenced by different matrix components, tensile properties were evaluated.

Mechanical tests took place at 50% relative humidity and 23 °C. The Young’s modulus (MPa), tensile strength (MPa), and elongation at break (%) were determined using an Instron 1000 N test machine (Norwood, MA, USA) operated at a test speed of 10 mm/min.

### 2.11. Scanning Electron Microscopy (SEM)

SEM microscopy was used to examine the surface morphology and microstructural uniformity of the films, revealing the distribution of fillers and matrix compatibility.

The SEM images were recorded using a VEGA TESCAN microscope equipped with a low-vacuum secondary electron detector, operating at an acceleration voltage of 20 kV and at room temperature. Samples were coated with a 10 nm gold layer prior to SEM imaging.

### 2.12. Anti-Inflammatory Activity of Materials

To evaluate the anti-inflammatory potential of the developed materials by measuring their ability to inhibit protein denaturation, an in vitro assay based on egg albumin denaturation was performed, as described by Ameena et al. [22], with a few modifications. Initially, a stock solution was prepared by mixing 40 mL of fresh egg albumin with 140 mL of phosphate-buffered saline solution (PBS, pH 6.4, 10 mM). The resulting mixture was then filtered to remove any particulate matter. From the filtered solution, 2 mL was taken and diluted with an additional 2 mL of PBS, followed by incubation at 35 °C for 30 min and heating at 70 °C for 5 min to induce denaturation. The appearance of opalescence was monitored as an indicator of protein denaturation. The absorbance of the solution was measured at 660 nm using the UV–Vis spectrophotometer, after cooling. This value represented the control sample for further comparisons.

The same procedure was applied to the analyzed materials, with the addition of 0.02 g of each sample to the prepared egg albumin solution. After incubation at 35 °C for 10 min, the solutions were filtered to remove any insoluble residues. The filtered solutions were then heated at 70 °C for 10 min to induce denaturation. The degree of inhibition of protein denaturation was calculated using Equation (2):

(2) $$Inhibition\;(\%)=\frac{A_{control}-A_{sample}}{A_{control}}\times 100$$

where *A_control_* and *A_sample_* are the absorbances of control and sample.

All measurements were performed in triplicate. This calculation allowed the determination of the percentage inhibition of denaturation for each sample.

### 2.13. In Vitro Biocompatibility (MTS Assay) of Materials

To assess the cytocompatibility of the films by determining the viability of human fibroblasts in contact with material extracts, the MTS assay was performed.

Human gingival fibroblasts (HGF, CLS Cell Lines Service GmbH, Eppelheim, Germany) were seeded (5 × 10^4^ cells/mL) into tissue culture-treated 96-well plates. The biocompatibility of obtained materials was assessed with the MTS assay, using the CellTiter 96^®^ AQueous One Solution Cell Proliferation Assay (Promega, Madison, WI, USA), according to the manufacturer’s instructions. Samples (10 mg/mL) were extracted over 24 h, at 37 °C, in a complete cell culture medium: MEMα medium (minimal essential medium Eagle—alpha modification) with 10% fetal bovine serum and 1% Penicillin–Streptomycin–Amphotericin B mixture (all from Gibco, Thermo Fisher Scientific, Waltham, MA USA). Cells were incubated with fresh complete medium (control) or diluted samples’ extracts (0.1/0.5/1 mg/mL) for 24 h. MTS absorbance at 490 nm was recorded on a FLUOstar^®^ Omega microplate reader (BMG LABTECH, Ortenberg, Germany). Experiments were performed in triplicate, and treated cell viability was expressed as a percentage of control cells’ viability (means ± standard deviation).

### 2.14. Swelling Test

Swelling test was used to determine the water absorption capacity and swelling kinetics of the films, which influence their hydration behavior and drug release profile.

For the swelling test, 0.04 g of the materials were immersed in distilled water at room temperature. Samples were collected at predetermined intervals ranging from 10 to 600 s. At each time point, the swollen sample was gently blotted with filter paper to remove excess surface water, weighed, and then returned to the water for continued swelling. This process was repeated until the swelling capacity reached equilibrium, indicated by no significant further increase in weight. Therefore, water absorbed (*g*/*g*) was calculated using Equation (3):

(3) $$Water\;absorbed\;(g/g)=\frac{W_{s}-W_{d}}{W_{d}}$$

where *W_s_* is weight of the swollen material at different time *t*, and *W*_*d*_ is the weight of the dry material.

The swelling profiles of the materials were analyzed using kinetic models, including first-order kinetics and pseudo-second-order kinetics (Equations (4) and (5)). These models were applied to assess and compare the swelling behavior quantitatively [23].

(4) $$\text{First order:}\;ln(Q_{e}-Q_{t})=ln(Q_{e})-k_{1}\times t$$

(5) $$\text{Pseudo-second order:}\;\frac{t}{Q_{t}}=\left(\frac{1}{k_{2}\times Q_{e}}\right)+\frac{t}{Q_{e}}$$

where *Q_e_* and *Q_t_* are defined as the swelling degree at equilibrium and the swelling degree at time (*t*), expressed in g water/g material. *k*_1_ and *k*_2_ are swelling kinetic constants.

### 2.15. In Vitro RC Extract Release Study

An in vitro release study was conducted to investigate the release kinetics of bioactive compounds from the films and identify the mathematical model that best describes the release mechanism of bioactive principles from RC extract.

The materials (0.1 g) were introduced in 25 mL distilled water at room temperature. At different time intervals, the solutions were analyzed using the UV–Vis spectrophotometer at 360 nm. The wavelength of 360 nm was selected based on the UV–Vis absorption maximum of RC extract in an aqueous solution, corresponding to the π–π* transitions of polyphenolic compounds (primarily flavonoids), which are known to absorb strongly in this region [10,11]. The concentration of RC extract was determined by using a calibration curve.

Release kinetics were analyzed by fitting the data to mathematical models, including Higuchi, Makoid–Banakar, Hixon–Crowell, Gompertz, and Korsmeyer–Peppas, to determine the release mechanism.

## 3. Results and Discussions

### 3.1. FTIR Spectra of Li and LiAs

**Figure 2:**
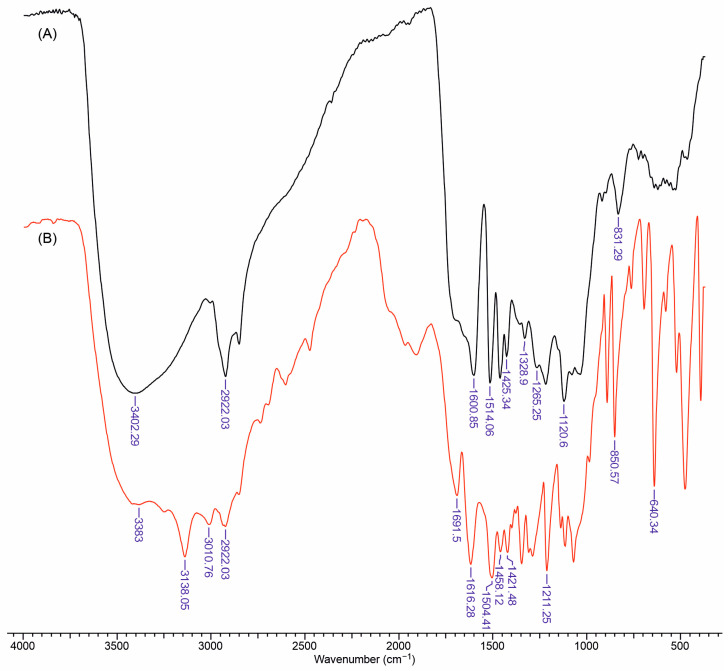
FTIR spectra of Li (**A**) and LiAs (**B**).

Figure 2A shows the characteristic FTIR profile of unmodified lignin, with a broad absorption band centered around 3400 cm^−1^ attributed to the stretching of phenolic and aliphatic hydroxyl groups, weaker signals near 2920 cm^−1^ and 2850 cm^−1^ from the aliphatic C–H stretches, and prominent aromatic ring vibrations at approximately 1600 cm^−1^, 1510 cm^−1^, and 1425 cm^−1^. The peaks at 1504, and 1421 cm^−1^ are related to the ring-stretch vibrations in lignin [24].

The band at about 1265 cm^−1^ is typically assigned to guaiacyl-type ring breathing or C–O stretching, while additional peaks below 900 cm^−1^ arise from out-of-plane aromatic C–H deformations. Figure 2B, representing lignin esterified with aspartic acid, retains many of the same aromatic bands but displays new features that signify the formation of ester or amide linkages and the incorporation of nitrogen-containing functional groups. A more pronounced carbonyl absorption emerges near 1700 cm^−1^, which can be associated with ester and/or amide C=O stretches introduced by the reaction between lignin’s hydroxyl groups and aspartic acid’s carboxyl functionalities. In addition, an increased intensity in the broad region around 3010 and 3138 cm^−1^ may reflect overlapping N–H stretches [25,26,27], while subtle shifts in the aromatic ring bands suggest a partial modification of lignin’s phenolic moieties. These spectral differences provide clear evidence that aspartic acid has been successfully grafted onto the lignin structure through esterification, yielding a modified lignin with both a retained aromatic character and newly formed nitrogenous linkages.

### 3.2. ^13^C Spectra of Li and LiAs

**Figure 3:**
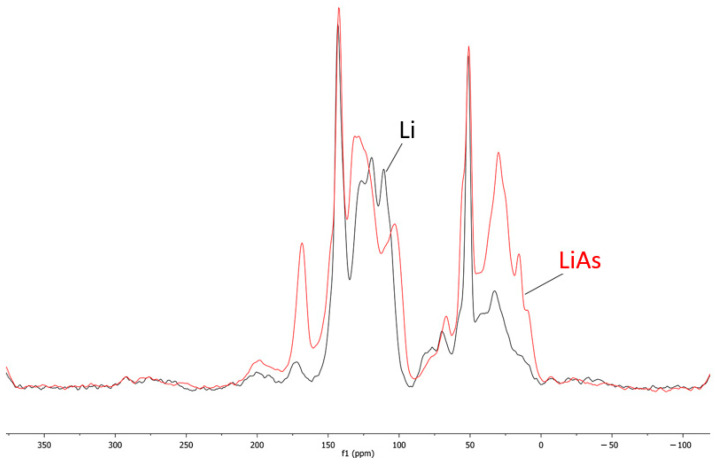
^13^C spectra of Li and LiAs.

In the ^13^C NMR spectra (Figure 3), the unmodified lignin (Li) and the lignin esterified with aspartic acid (LiAs) both exhibit the broad, overlapping signals characteristic of lignin’s heterogeneous structure, yet several key differences confirm esterification. In Li, the aromatic envelope extends from roughly 110 to 160 ppm, reflecting guaiacyl and syringyl units that often give rise to resonances near 114–115, 119–120, 131–133, and 147–148 ppm, and the methoxyl groups appear around 55–57 ppm. The aliphatic side chain region of unmodified lignin generally spans 50–90 ppm, encompassing α-, β-, and γ-carbon signals of the various lignin interunit linkages.

In the LiAs spectrum, the aromatic signals remain but show minor chemical shift changes and intensity redistribution, indicating alterations in the electronic environment as new substituents are introduced. More pronounced are the emergence and shifts in the aliphatic region, particularly between 30 and 60 ppm [28], where additional resonances arise from the α- and β-carbons of the aspartic acid moiety. The most definitive evidence of ester formation is observed in the carbonyl region, around 170–180 ppm, where new and enhanced signals signify ester or amide linkages involving the aspartic acid carboxyl groups. Collectively, these spectral differences, including new aliphatic peaks due to aspartic acid, intensified resonances in the ester/amide carbonyl domain, and subtle shifts throughout the aromatic region prove the successful covalent attachment of aspartic acid onto the lignin backbone.

### 3.3. XPS Spectra of Li and LiAs

**Figure 4:**
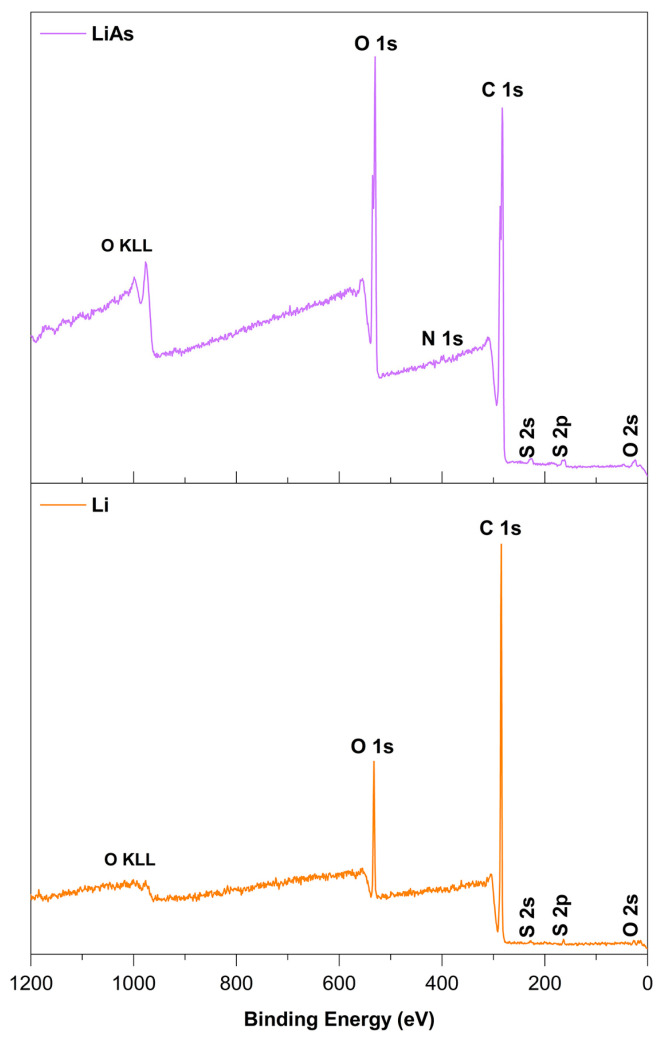
XPS spectra of Li and LiAs.

X-ray photoelectron spectroscopy (XPS) was employed to investigate the elemental composition and chemical functionalities of lignin (Li) and lignin esterified with aspartic acid (LiAs). The survey spectra (Figure 4) confirm that both materials are predominantly composed of carbon (C) and oxygen (O), with minor contributions from sulfur (S). The Lignoboost lignin (Li) sample exhibited relative atomic concentrations of 77.50% C, 21.64% O, and 0.85% S (Table 2), consistent with previously reported values for technical lignins.

The high-resolution deconvolution of the O 1s spectrum of Li (Table 3) revealed three primary contributions: the peak at 531.9 eV (10.01%) was attributed to oxygen in carbonyl environments (C=O, such as ketones or quinones), the dominant peak at 533.3 eV (76.98%) corresponded to hydroxyl and ether functionalities (C–OH/C–O), and the peak at 534.3 eV (13.01%) was associated with peroxide or adsorbed oxygen species (O–O) [29].

Similarly, the C 1s spectrum of Li (Table 4) exhibited signals at 285.0 eV (C–C/C–H, 57.94%), 286.5 eV (C–O/C–OH, 35.24%), 287.5 eV (C=O, 5.33%), and 289.0 eV (O–C=O, 1.49%) [13]. These assignments reflect the structural complexity of lignin, which includes aromatic rings, aliphatic chains, and a variety of oxygenated functionalities.

Upon esterification with aspartic acid, the XPS data for LiAs indicated clear chemical modifications. The appearance of a N 1s peak at 398.0 eV in the LiAs spectrum (Table 5) confirmed the incorporation of nitrogen, a signature of the amino acid moiety. The O 1s peak in LiAs, centered around 531.0 eV, showed increased intensity and relative concentration (22.98%) compared to Li, suggesting a higher content of carbonyl-containing groups—likely ester and amide functionalities introduced during the reaction. The C 1s spectrum of LiAs also shifted, showing increased intensity at 286.0 eV, indicative of an enhanced presence of oxygenated carbons (e.g., C–O, C–N, C=O).

Together, these spectroscopic changes, particularly the rise in carbonyl-associated oxygen signals and the introduction of nitrogen, strongly support the successful esterification of lignin with aspartic acid. These findings are in agreement with complementary FTIR and ^13^C NMR analyses and confirm the structural transformation of Li into LiAs through the formation of ester and amide linkages.

### 3.4. FTIR Spectra of RC Extract

Based on previously published data [10,11,12,21], the aqueous extract of *Rosa canina* is known to contain significant levels of flavonoids (quercetin, rutin, catechin), phenolic acids (gallic and ellagic acid), and vitamin C. These compounds are responsible for the extract’s anti-inflammatory, antioxidant, and skin-repairing effects. The extraction method applied in this study was designed to retain these hydrophilic bioactives, which were confirmed through total phenolic and flavonoid assays.

**Figure 5:**
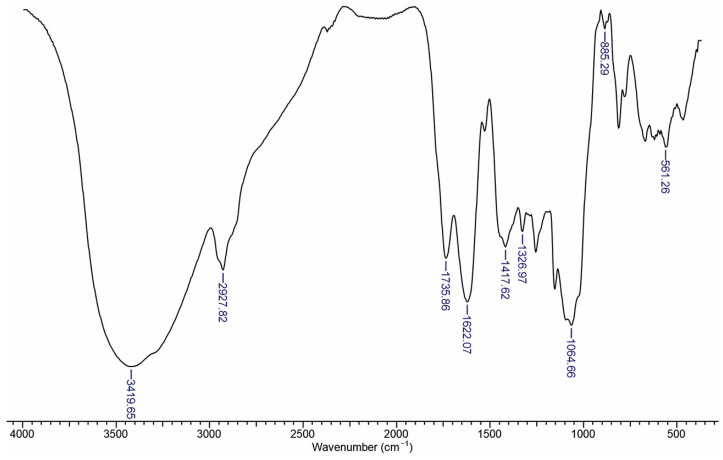
FTIR spectra of RC extract.

The FTIR spectrum of *Rosa canina* aqueous extract is dominated by a broad absorption band extending from approximately 3200 cm^−1^ to 3600 cm^−1^, characteristic of O–H stretching vibrations in both water and phenolic compounds (Figure 5). The slight shoulder or variations within this region suggest diverse hydrogen-bonding environments, likely associated with polyphenols, organic acids, and other hydroxyl-rich components in the extract.

A weaker band or shoulder around 2900 cm^−1^ can be attributed to C–H stretching modes of aliphatic moieties. Around 1600–1650 cm^−1^, there is a strong absorption often correlated with aromatic C=C stretching or conjugated C=O groups from phenolics, while any shoulders near 1700 cm^−1^ would point to carbonyl functionalities such as those found in carboxylic acids or esters. Peaks at 1735, 1622, and 1417 cm^−1^ represent symmetric carbonyl group (C=O) stretching vibrations in ketones, aldehydes, and carboxylic acids, C=C stretching vibrations from aromatic rings, and C–OH stretching vibrations, respectively. The peaks from the region of 1326 to 1064 cm^−1^ are associated with the C–O stretching from of esters groups. Peaks at 885 cm^−1^ and 561 cm^−1^ correspond to δ C-H in-plane deformation vibrations [12,30].

Collectively, these features confirm the presence of both phenolic constituents and carbohydrate-like or glycosidic components in the aqueous extract, consistent with the expected phytochemical composition of *Rosa canina*.

### 3.5. FTIR Spectra of Materials

**Figure 6:**
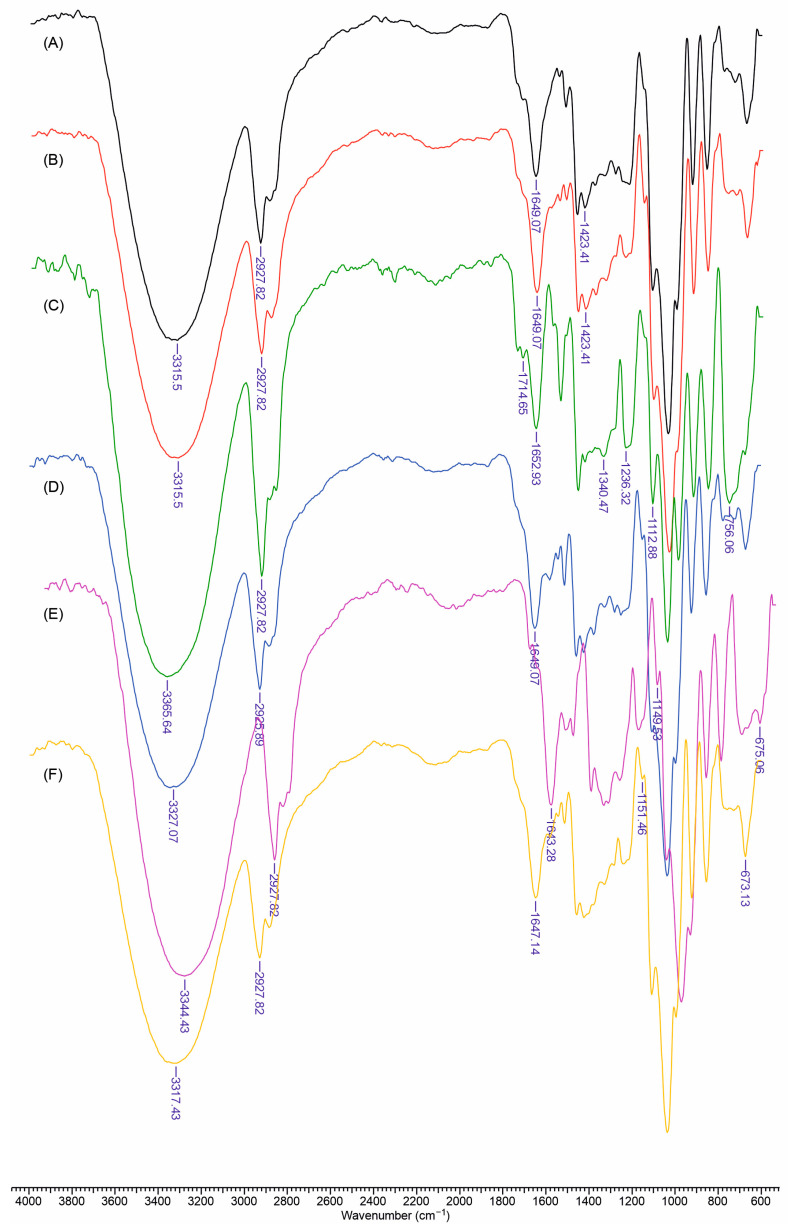
FTIR spectra of obtained materials: (**A**) Gu/DexS; (**B**) Gu/DexS/RC; (**C**) Gu/DexS/Li; (**D**) Gu/DexS/Li/RC; (**E**) Gu/DexS/LiAs; (**F**) Gu/DexS/LiAs/RC.

The FTIR spectra of the Gu/DexS formulations, both with and without the incorporation of lignin, esterified lignin, and *Rosa canina* extract, reveal a series of characteristic absorption bands that provide detailed insights into the chemical composition and molecular interactions within these composites (Figure 6). All samples exhibit a broad band in the 3300–3400 cm^−1^ region, which is attributed to O–H stretching vibrations arising from polysaccharides, phenolic groups, and residual moisture. In the Gu/DexS and Gu/DexS/RC samples, the pronounced nature of this band indicates an extensive hydrogen bonding network, with the addition of *Rosa canina* extract further modulating these interactions through its polyphenolic hydroxyl groups.

Complementary to this, absorptions near 2920 cm^−1^ are observed, corresponding to the C–H stretching vibrations of the polysaccharide backbone, while a band between 1640 and 1650 cm^−1^ is ascribed to bound water and potential overlap with amide or aromatic ring stretching vibrations. Notably, the DexS-containing matrices display a distinct set of peaks between 1200 and 1250 cm^−1^, which arise from the asymmetric stretching of sulfate groups.

Upon the incorporation of lignin—either in its native form or esterified with aspartic acid—additional spectral features emerge. Samples containing lignin display intensified signals in the 1500–1600 cm^−1^ region, which are consistent with aromatic ring vibrations inherent to lignin (Figure 6C,D). Moreover, in formulations with esterified lignin, more pronounced bands near 1700–1740 cm^−1^ are evident, reflecting the formation of new ester and carboxylic functionalities introduced through the reaction with aspartic acid (Figure 6E,F). The presence of *Rosa canina* extract further enhances the intensity of the broad O–H band, underscoring the contribution of extra hydroxyl groups from its polyphenolic compounds. In these RC-containing formulations, an additional signal between 1716 and 1741 cm^−1^ is observed and assigned to symmetric C=O stretching vibrations in ketones, aldehydes, and carboxylic acids (Figure 6B,D,F).

Additional absorption features further delineate the complex chemical environment within these materials. The spectra reveal O–H stretching at around 3340 cm^−1^, C–H stretching of CH_2_ groups between 2850 and 2927 cm^−1^, and C–H bending vibrations occurring from 1320 to 1425 cm^−1^, alongside C–C stretching near 1200 cm^−1^. Specific carbohydrate linkages are also identified, with bands at 870 cm^−1^ and 930 cm^−1^ corresponding to 1–4 and 1–6 linkages of galactose and mannose, respectively [31]. Moreover, two characteristic bands at 1236 cm^−1^ and 820 cm^−1^ are attributed to the asymmetric stretching of sulfate groups in ester sulfate and the symmetric stretching of C–O–SO_3^−^_ groups in the DexS structure, respectively [32]. Subtle shifts and changes in intensity within the fingerprint region (900–1200 cm^−1^) further suggest interactions among the hydroxyl, carboxyl, and sulfate groups of the matrix and the phenolic or carbonylic functionalities of the extract.

Overall, the FTIR analysis confirms the successful incorporation of both native and esterified lignin as well as *Rosa canina* extract into the Gu/DexS-based formulations. The observed vibrational changes provide valuable insight into the chemical modifications and intermolecular interactions within these composite materials, thereby contributing to a comprehensive understanding of their structural properties.

### 3.6. Density of Materials

**Figure 7:**
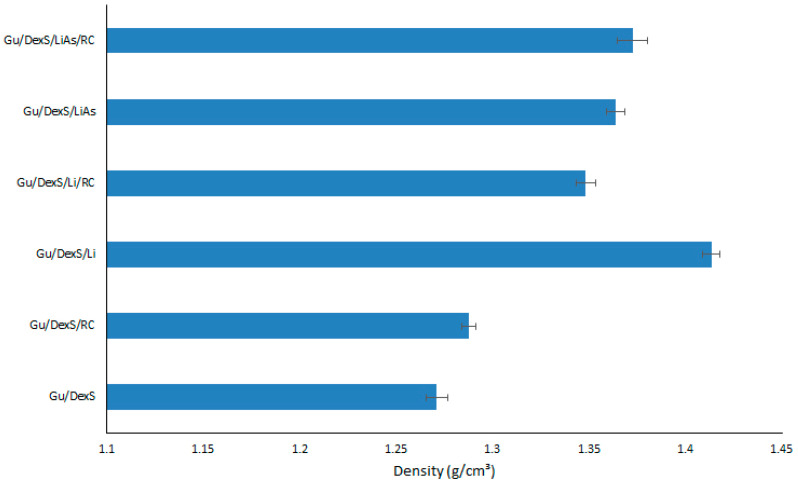
The density of materials.

The densities of the obtained materials are presented in Figure 7. In general, when fillers are added to a polymeric matrix, the material’s density often increases because fillers are typically denser than the polymeric matrix itself. Additionally, fillers can enhance the packing efficiency within the material, reducing void spaces and making the composite more compact. This increase in density affects the material’s mechanical properties, including strength or rigidity [33].

Material containing Li (Gu/DexS/Li) presented the highest density (1.41 g/cm^3^) due to the inherent properties of lignin itself. Lignin is a complex, high-molecular-weight biopolymer, with a relatively dense and rigid structure compared to other organic polymers. The aromatic rings and phenolic groups from the Li structure [34] contribute to a higher molecular mass and stronger intermolecular forces, making it more compact and less flexible.

Materials containing LiAs (Gu/DexS/LiAs and Gu/DexS/LiAs/RC) exhibit a slight decrease in density (1.36 and 1.37 g/cm^3^) compared to those containing unmodified Li (Gu/DexS/Li and Gu/DexS/Li/RC with density values of 1.41 and 1.34 g/cm^3^). This decrease in density can be logically attributed to the chemical and structural alterations introduced during the esterification process.

Lignin is inherently a dense, aromatic biopolymer, characterized by rigid phenylpropanoid units and extensive intermolecular interactions, including π-π stacking and hydrogen bonding. These features contribute to a tightly packed and compact molecular arrangement. However, the esterification of lignin with aspartic acid disrupts this native architecture. The addition of aspartic acid introduces bulky, hydrophilic side chains that increase steric hindrance and reduce the degree of molecular packing. This structural modification not only interferes with aromatic stacking but also introduces greater conformational flexibility and spatial irregularity within the polymer network.

Furthermore, the enhanced polarity of the LiAs-modified structure may facilitate the retention of small amounts of water or promote the formation of microvoids, both of which can contribute to a lower overall material density. Thus, the observed reduction in density for LiAs-based composites is consistent with the molecular reorganization and increased free volume induced by esterification, highlighting the significant impact of chemical modification on the physical properties of lignin-based systems.

The presence of RC extract also influences material density. Gu/DexS/RC materials present a lower density (1.28 g/cm^3^) compared to Gu/DexS/Li. RC extract consists of bioactive compounds [35], which have a lower molecular weight and are less dense than lignin’s rigid, aromatic polymer structure. These materials disrupt the packing efficiency of the polymeric matrix, resulting in a less dense overall structure.

### 3.7. Swelling Behavior of Materials

**Figure 8:**
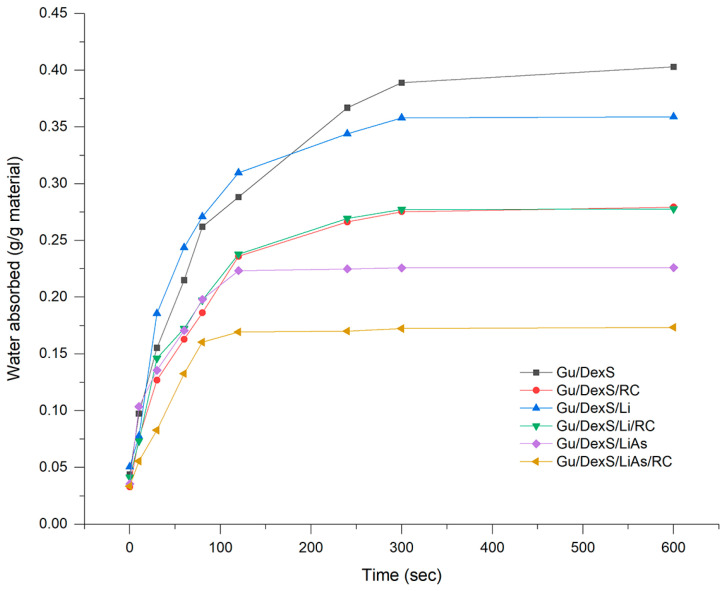
The swelling ratio of the materials.

The swelling behavior of materials has been shown to significantly influence the release profile of RC extract, highlighting the interplay between material dynamics and release mechanisms. The system reaches a swelling equilibrium (after 600 s) (Figure 8) when the solvent diffusion balances the resistance offered by the matrix structure and fillers.

The Gu/DexS material, being highly hydrophilic, ensures significant distilled water absorption (0.40 g/g), but the presence of Li, LiAs, and RC extract reduces the maximum swelling capacity (0.35 g/g, 0.27 g/g, and 0.23 g/g) by occupying free spaces in the polymeric matrix. In the early stages, water molecules rapidly diffuse into the matrix due to the hydrophilic nature of Gu (rich in –OH groups) and the anionic DexS. Being hydrophobic, Li and LiAs act as a barrier to solvent penetration, slowing down the diffusion process and modifying the rate of swelling. RC extract influences the swelling behavior based on its interaction with the polymer matrix. It forms strong intermolecular interactions (hydrogen bonds) and reduces the swelling rate by reinforcing the matrix structure [36].

Based on the models for swelling kinetics, the pseudo-second-order kinetic fitted well to the swelling process for all the obtained materials (Table 6, Figure 8).

The correlation between swelling kinetics and the release profile of RC extract is evident in the interplay between the pseudo-second-order swelling model and the Korsmeyer–Peppas release model. The pseudo-second-order kinetics indicate that swelling is governed by specific interactions between the polymeric matrix (Gu/DexS) and the swelling medium, resulting in a controlled and gradual expansion of the material. The Korsmeyer–Peppas model, characterized by its diffusion coefficient, n, suggests an anomalous transport mechanism where both diffusion and polymer relaxation contribute to the release process. The controlled swelling rate, which decreases as the equilibrium is approached, aligns with the quasi-Fickian release behavior described by the model, ensuring a gradual and sustained release.

The material composition, including the hydrophilic matrix and the fillers, further influences these processes, supporting a release mechanism that balances swelling-driven matrix expansion and diffusion-mediated transport, making the system ideal for skin applications.

### 3.8. Mechanical Properties of Materials

**Figure 9:**
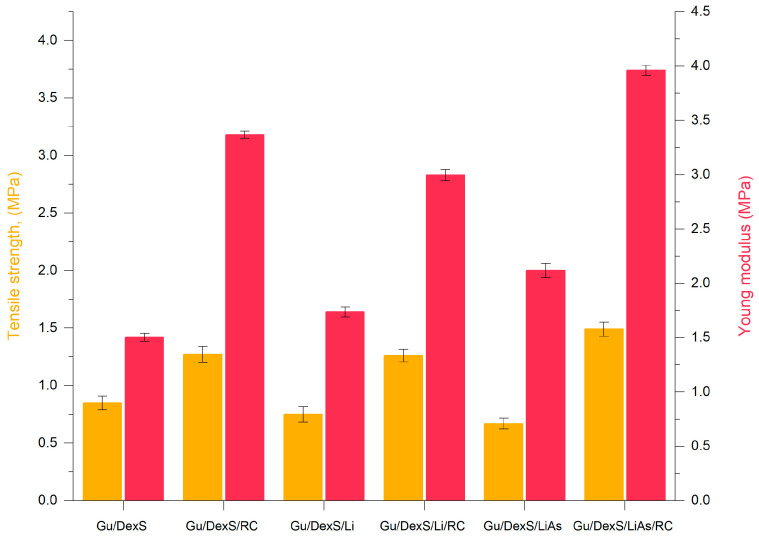
The tensile strength of the obtained materials. Graphical data were expressed as means ± standard deviation.

The mechanical properties of the Gu/DexS-based materials exhibit significant variations depending on the presence of lignin (Li), esterified lignin (LiAs), and *Rosa canina* (RC) extract, as observed in the tensile strength and Young’s modulus evaluations (Table 7, Figure 9).

The base formulation, Gu/DexS, serves as a reference, displaying a tensile strength of approximately 1.0 MPa and a Young’s modulus of 1.42 ± 0.15 MPa. The incorporation of RC extract (Gu/DexS/RC) results in a considerable enhancement, increasing the tensile strength to about 1.3 MPa and elevating the Young’s modulus to 3.18 ± 0.96 MPa. This represents an increase of over 120%, which suggests that the polyphenolic compounds in RC contribute to intermolecular hydrogen bonding within the matrix, reinforcing the structural integrity of the film. The observed improvement is consistent with previous studies reporting increased tensile strength and antioxidant activity upon the introduction of polyphenol-rich grape seed and green tea extracts [37].

The addition of lignin (Gu/DexS/Li) further improves mechanical performance, with Young’s modulus increasing to approximately 1.7 MPa and tensile strength reaching 1.5 MPa. Lignin molecules tend to form particle-like structures that contribute to a more rigid phase within the polymeric matrix, strengthening the network and enhancing stiffness and tensile strength [38,39]. Consequently, the Gu/DexS/Li material exhibits an 18.26% reduction in elongation at break compared to Gu/DexS, which is indicative of its increased stiffness. A more pronounced effect is observed when lignin is combined with RC extract (Gu/DexS/Li/RC), where tensile strength increases to 1.8 MPa and Young’s modulus reaches 2.83 ± 0.19 MPa, confirming a synergistic effect between lignin and polyphenols that further reinforces the composite through additional hydrogen bonding.

A substantial shift in mechanical behavior is observed upon the esterification of lignin with aspartic acid (LiAs). The Gu/DexS/LiAs formulation exhibits an increase in rigidity by approximately 30–40%, leading to a tensile strength of 0.67 ± 0.32 MPa and a significantly reduced elongation at break of 203.4 ± 10.9% (Table 7). These findings can be attributed to electrostatic interactions between the negatively charged aspartate groups of lignin ester and the polymeric matrix, which contains negatively charged sulfate groups from DexS. The increased rigidity is further reflected in the Young’s modulus of 2.2 MPa, marking a 45% improvement compared to Gu/DexS.

The most pronounced enhancement in mechanical properties is observed when both esterified lignin and *Rosa canina* extract are present in the Gu/DexS/LiAs/RC formulation. This composite exhibits a tensile strength of 1.49 ± 0.17 MPa, which is more than double that of Gu/DexS/LiAs alone, and a Young’s modulus of 3.74 ± 0.08 MPa, representing an overall increase of over 160% relative to Gu/DexS. The enhanced stiffness can be explained by the combined effects of additional hydrogen bonding between the hydroxyl groups of the matrix and the polyphenolic compounds in RC, as well as the increased crosslinking capacity introduced by esterified lignin. Previous studies have suggested that such polyphenolic-rich extracts contribute to the formation of denser polymer networks via interfacial adhesion, further reinforcing mechanical stability [40].

The mechanical performance of the developed Gu/DexS-based materials was evaluated in comparison with the literature data on polysaccharide-based hydrogels designed for dermal or biomedical use. The optimized formulation of Gu/DexS/LiAs/RC exhibited a tensile strength of 1.49 MPa and an elongation at break of 237.8%, values significantly superior to those typically reported for commercial hydrogel dressings, which range between 0.03–0.3 MPa for tensile strength and 10–100% for elongation at break, depending on composition and crosslinking density [27,31,34]. These results indicate that the incorporation of both lignin and *Rosa canina* extract led to enhanced polymer chain interactions, improving both the resistance to mechanical stress and the elasticity of the final hydrogel network. The obtained mechanical parameters suggest that these hybrid materials can meet or exceed the structural requirements of current skin-compatible biomaterials, and may be particularly suitable for flexible wound coverings or dermal patches.

Overall, the results confirm that, while lignin alone provides a moderate increase in mechanical properties due to its role in crosslinking and network stabilization, the combination of esterified lignin and RC extract leads to a significantly stronger and stiffer material. The synergistic effect of these components enhances tensile properties, making the resulting biocomposite a promising candidate for applications requiring robust mechanical performance.

### 3.9. Material Morphology

The SEM images provide valuable insights into the morphological characteristics of the Gu/DexS-based materials and their correlation with the mechanical properties and component interactions.

**Figure 10:**
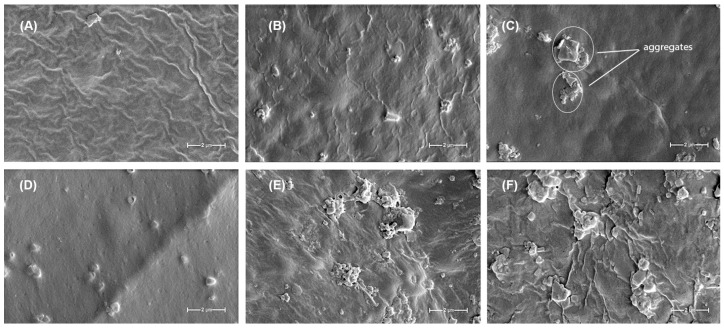
The SEM micrographs of the obtained materials (magnification ×10,000): (**A**) Gu/DexS; (**B**) Gu/DexS/RC; (**C**) Gu/DexS/Li; (**D**) Gu/DexS/Li/RC; (**E**) Gu/DexS/LiAs; (**F**) Gu/DexS/LiAs/RC.

The reference material, Gu/DexS (Figure 10A), exhibits a relatively smooth and homogeneous surface with slight wrinkles, indicative of an organized polymeric network. This morphology is consistent with the moderate mechanical strength observed for this formulation, where the polysaccharide-based matrix provides a uniform structure but lacks reinforcement.

Upon the incorporation of *Rosa canina* extract (Gu/DexS/RC, Figure 10B), the surface becomes slightly rougher, with minor agglomerations and irregularities. This suggests the integration of polyphenolic compounds, likely through hydrogen bonding interactions with the polymeric matrix. These interactions lead to an increase in Young’s modulus and tensile strength, as previously observed, reinforcing the film without significantly disrupting its homogeneity.

The addition of lignin (Gu/DexS/Li, Figure 10C) results in a noticeable change in surface morphology, characterized by increased roughness and the appearance of distinct particulate aggregates, as highlighted in the image. These aggregates reflect localized clustering of lignin within the polymer matrix, which likely contributes to the observed enhancement in mechanical strength. While this structural reinforcement improves tensile resistance, the heterogeneous distribution of these domains may also introduce local stress concentrations, potentially explaining the moderate decrease in elongation at break compared to the lignin-free formulation.

When both lignin and *Rosa canina* extract are present (Gu/DexS/Li/RC, Figure 10D), the surface appears somewhat smoother compared to Gu/DexS/Li but retains small dispersed particles. This suggests a better dispersion of lignin within the matrix, likely facilitated by interactions with the polyphenolic compounds from the extract. The mechanical properties reflect this synergy, as the material exhibits improved tensile strength and modulus while maintaining a more balanced structural integrity.

A major shift in morphology is observed in the Gu/DexS/LiAs sample (Figure 10E), where large agglomerates and morphological heterogeneity become evident. The esterification of lignin with aspartic acid introduces additional functional groups, leading to stronger electrostatic interactions with the negatively charged DexS matrix. This results in a denser and stiffer structure, as evidenced by the significant increase in Young’s modulus. However, the reduced elongation at break and breaking length indicate that this enhanced rigidity compromises flexibility, leading to a more brittle material.

The most heterogeneous morphology is observed in the Gu/DexS/LiAs/RC sample (Figure 10F), where large irregular aggregates are dispersed throughout the matrix. This suggests strong interactions among the esterified lignin, *Rosa canina* extract, and the polysaccharide matrix, leading to significant reinforcement and increased morphological heterogeneity. The mechanical properties confirm this trend, with the highest Young’s modulus and breaking length values, indicating a rigid and mechanically robust material. However, the excessive aggregation may contribute to localized stress points, which could affect the material’s overall flexibility.

Overall, the SEM analysis confirms that lignin, esterified lignin, and *Rosa canina* extract interact differently with the Gu/DexS matrix, affecting both mechanical properties and morphology. While lignin and RC extract improve mechanical performance through hydrogen bonding and dispersion effects, the esterification of lignin with aspartic acid introduces strong electrostatic interactions that lead to a denser but more brittle structure. The combination of esterified lignin and RC extract results in the most rigid material, confirming that chemical modifications and polyphenol interactions play a critical role in determining the final properties of the composite films.

### 3.10. RC Extract Release from Materials

The mathematical modeling of drug release from polymeric matrices based on natural polysaccharides provides a framework to understand and predict the underlying transport mechanisms. The Higuchi model [41], one of the earliest and most widely used, is typically expressed as Equation (6):

(6) $$Q=k_{H}\times \sqrt{t}$$

where *Q* is the amount of drug released at time *t*, and *k_H_* is the Higuchi dissolution constant. This parameter *k_H_* combines characteristics of drug diffusivity, solubility, and matrix porosity, indicating how rapidly the drug migrates out of the polymer. When the release kinetics follow Fickian diffusion and the matrix remains relatively intact, a good fit to the Higuchi equation implies that diffusion is the dominant process controlling drug release.

In cases where the release profile exhibits a sigmoidal shape due to variable rates of swelling or erosion in natural polysaccharide matrices, the Makoid–Banakar model [42] can be applied. It is generally written as Equation (7):

(7) $$F(t)=a\times {\left(1-e^{-kt}\right)}^{n}$$

where *F*(*t*) is the fraction of drug released, *a* is a scaling factor related to the maximum release, *k* is the release rate constant, and *n* denotes the shape of the release curve. Here, *a* reflects the asymptotic extent of release, *k* provides insight into how quickly the transition from slow to accelerated release occurs, and *n* controls the steepness or sigmoid form of the curve. These parameters become especially relevant in natural polysaccharide systems that may exhibit an initial lag phase followed by a distinct acceleration in drug release once the polymer begins to swell or degrade.

The Weibull model [43] is a versatile mathematical tool widely employed to describe the kinetics of drug release from polymeric matrices. Its flexible form allows for the characterization of various release mechanisms, ranging from diffusion-dominated processes to degradation-controlled systems. The general equation (Equation (8)) of the Weibull model is as follows:

(8) $$y=A\times \left(1-e^{-{\left(k\times \left(x-x_{c}\right)\right)}^{d}}\right)$$

where *y*—cumulative drug release at time *x*; *A*—maximum cumulative release (asymptotic value); *k*—release rate constant, indicating the speed of drug release; *x_c_*—lag time before significant drug release begins; *d*—shape parameter, which determines the nature of the release curve.

By fitting experimental data to the Weibull model, the values of *A*, *k*, *x_c_*, and *d* provide valuable insights into the drug release profile: *A*—indicates the total amount of drug that can be released; *k*—reflects the speed of release, influenced by the matrix’s structure and drug properties; *x_c_*—identifies any delay in release, such as a hydration phase or lag due to matrix swelling; *d*—reveals the dominant release mechanism, distinguishing between diffusion and matrix degradation.

The model accommodates various types of polymeric matrices, including hydrogels, crosslinked networks, and composite materials. Its adaptability allows it to describe systems with single or combined release mechanisms.

The Weibull model is often used to fit experimental release data, enabling the determination of the kinetics and mechanisms of drug release. For example, a low *d* value (<1) indicates diffusion-dominated release, common in hydrophilic polymeric matrices.

By comparing *k* and *d* values across different formulations, researchers can evaluate the influence of polymer composition, crosslinking density, and drug–polymer interactions on release behavior.

When matrix erosion or structural disintegration governs release rather than diffusion alone, the Hixson–Crowell cube-root model [44] is often appropriate. It is typically expressed as Equation (9):

(9) $$Q_{0}^{1/3}-Q^{1/3}=k_{HC}\times t$$

where *Q*_0_ is the initial amount of drug in the dosage form, *Q* is the remaining amount of drug at time *t*, and *k_HC_* is the Hixson–Crowell constant; this equation underscores how changes in surface area drive drug liberation. The constant *k_HC_* represents the rate at which the dosage form diminishes in size, capturing how quickly erosion of the polysaccharide matrix exposes a fresh surface area for drug release.

Another sigmoidal model often used for complex release kinetics is the Gompertz function, written in the dissolution context as Equation (10):

(10) $$F(t)=1-e^{-be^{\alpha t}}$$

where *b* and *α* determine the shape and rate of the release curve. The parameter *b* generally signifies the scale of the release process, sometimes interpreted as relating to the position of the inflection point, whereas *α* influences how quickly the release curve rises after an initial lag phase. When analyzing natural polysaccharide matrices, changes in polymer swelling or crosslinking can manifest in shifts in *b* and *α*, thereby indicating whether the system is prone to early rapid swelling or a delayed onset of drug release.

A versatile semi-empirical model is the Korsmeyer–Peppas equation [45], usually given as Equation (11):

(11) $$F(t)=k_{KP}\times t^{n}$$

where *k_KP_* is the release rate constant, and *n* is the release exponent. This exponent *n* classifies the release mechanism: if *n* ≈ 0.5, Fickian diffusion is typically dominant; if *n* is closer to 1.0, erosion or polymer relaxation strongly influences the release; values in between often indicate anomalous transport. In many natural polysaccharides that can undergo swelling and partial erosion, the Korsmeyer–Peppas model is invaluable for distinguishing which process—diffusion, swelling, or erosion—takes precedence. The constant *k_KP_* then provides a measure of how quickly the drug traverses the matrix, guiding formulation scientists in adjusting polymer properties to achieve the desired release rate.

Collectively, these models and their parameters depict how the microstructure of natural polysaccharides, as well as their swelling, erosion, and diffusion characteristics, influence the course of drug release. By comparing experimental dissolution profiles with predicted curves and identifying which parameters shift in response to formulation changes, it becomes possible to design polymeric systems with optimized release patterns suitable for a wide range of therapeutic applications.

To identify the most appropriate mathematical model describing the release of *Rosa canina* extract from the polymeric materials (Gu/DexS/RC, Gu/DexS/Li/RC, and Gu/DexS/LiAs/RC), the abovementioned mathematical models were evaluated. Model performance was compared using both the reduced chi-square (*red-χ*^2^) and the coefficient of determination (R^2^) as shown in Table 8.

Overall, the Makoid–Banakar model exhibited the most consistent goodness of fit across all three tested materials. In the Gu/DexS/Li/RC system, for instance, it yielded a reduced chi-square of 1.8442 and an R^2^ of 0.98799, outperforming the Weibull model (*red-χ*^2^ = 2.14893, R^2^ = 0.98694), as well as the other four models tested. In the Gu/DexS/LiAs/RC system, the Makoid–Banakar model again provided the best fit (*red-χ*^2^ = 2.19185, R^2^ = 0.99221), with notably lower chi-square and higher R^2^ compared to the next-best alternative (Weibull). Although Weibull demonstrated a slightly lower *red-χ*^2^ for Gu/DexS/RC (3.77609 vs. 5.65783 for Makoid–Banakar), Makoid gave consistently high R^2^ values (0.99292 in Gu/DexS/RC and above 0.98 in the other two systems), leading to superior overall performance when all samples were evaluated collectively.

Furthermore, a rank-sum approach was used to account for performance differences among the three systems. Each model was ranked based on its *red-χ*^2^ in each system; then, the ranks were summed. Makoid had the lowest total rank sum (i.e., it was either first or second place in each polymeric material), thus reinforcing its robust and reliable fit across all formulations. Given these findings, and considering that the Makoid–Banakar model uses a moderate number of parameters (which helps mitigate overfitting risks), Makoid was selected as the single most appropriate model to describe release kinetics from the three polymeric materials in this study.

**Figure 11:**
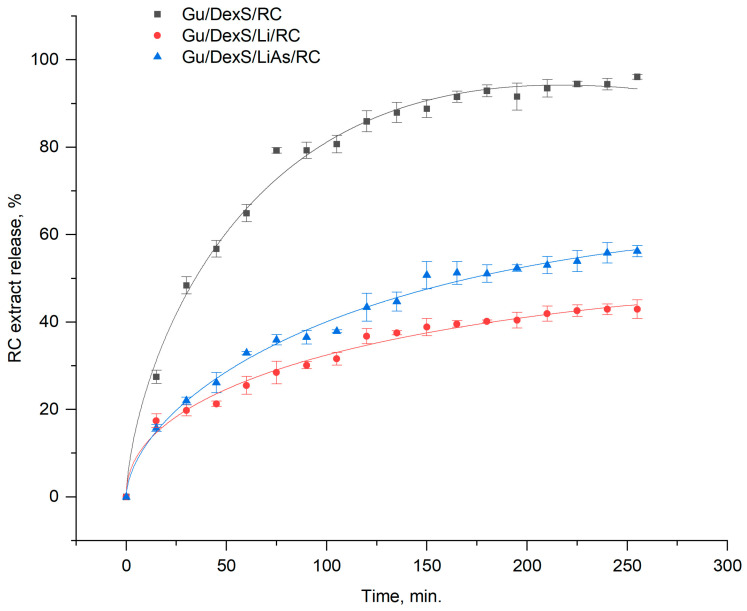
Release kinetic of RC extract from tested materials according to Makoid–Banakar model.

The release kinetic of RC extract from tested materials according to the Makoid–Banakar model is depicted in Figure 11.

In the Gu/DexS/RC formulation, the Makoid–Banakar parameters were *k* ≈ 5.84, *a* ≈ 0.00293, and *n* ≈ 0.635, indicative of a relatively brisk initial release (higher *k*) and a moderate exponent (*n*) that underlines both diffusion-driven kinetics and matrix relaxation effects. When lignin was incorporated into the Gu/DexS blend (Gu/DexS/Li/RC), *k* dropped by about 25% (to ≈4.38), reflecting a slower initial rate of RC extract release. The exponential decay factor *a* also showed a marked reduction of roughly 74% (to ≈0.00076), suggesting a more sustained deceleration of release over time. This behavior can be attributed to the increased hydrophobicity and structural density introduced by lignin, which limits water penetration and thus dampens the overall release rate. Concomitantly, the exponent *n* shifted to ≈0.451, highlighting a stronger reliance on diffusion processes relative to polymer relaxation, as the lignin-containing network restricts chain mobility.

Further modifications in the matrix composition were evident in Gu/DexS/LiAs/RC, where the esterification of lignin with aspartic acid altered the release profile once again. The rate constant *k* fell to ≈3.24, a clear sign that the composite became even less permissive to rapid RC release, yet *a* rose to ≈0.00123, partially restoring the influence of exponential decay. This indicates that the aspartic acid moieties, with their polar groups, somewhat balance out lignin’s hydrophobic character, allowing moderate re-swelling or structural reorganization. The exponent *n* remained close to 0.57, suggesting that the combined effects of diffusion and matrix relaxation persisted but were tempered by the interplay of the various components in the polymeric network. Altogether, the numerical values of *k*, *a*, and *n* show that Gu and DexS alone support faster and somewhat diffusion-limited release, while the addition of lignin slows the initial release and prolongs drug availability, and lignin-aspartic acid conjugates introduce intermediate hydrophilic–hydrophobic interactions that modulate both the rate and mechanism of release. The ability of the Makoid–Banakar model to incorporate a power-law component and an exponential decay term, each corresponding to distinct release mechanisms, highlights its superior performance in describing how Gu, DexS, Li, LiAs, and RC interact within the polymeric network to govern the overall release process.

### 3.11. Anti-Inflammatory Activity of Materials

**Figure 12:**
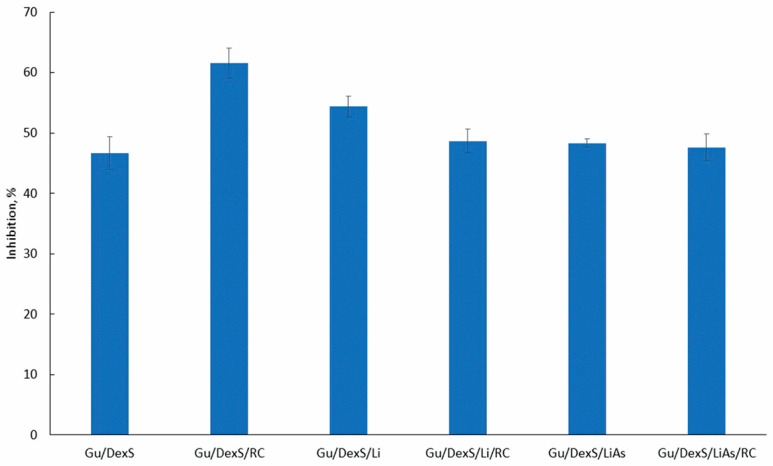
The anti-inflammatory activity of the obtained materials.

The anti-inflammatory activity of the Gu/DexS-based materials was evaluated using the egg albumin denaturation assay. The results (Figure 12) indicate that all formulations incorporating fillers exhibited the notable inhibition of protein denaturation (>45%). The strongest activity was recorded for Gu/DexS/RC (61.58%), followed by Gu/DexS/Li (54.36%) and Gu/DexS/Li/RC (48.70%), confirming the beneficial role of *Rosa canina* extract (RC) and lignin (Li) in mitigating protein aggregation under stress conditions.

The superior performance of Gu/DexS/RC is attributed to the phenolic and flavonoid content of RC extract (Section 2.4 and Section 2.5) that contributes to its antioxidant and anti-inflammatory properties [29]. The relatively smooth surface morphology of Gu/DexS/RC observed in the SEM micrographs (Figure 10B) supports the good dispersion of the extract in the matrix, facilitating homogeneous release and consistent surface bioactivity. Moreover, its moderate swelling capacity (0.23 g/g, Figure 8) supports rapid hydration, enhancing active compound diffusion to the application site.

Incorporating unmodified lignin (Gu/DexS/Li) also contributed to anti-inflammatory effects (54.36%) due to lignin’s intrinsic phenolic antioxidant activity [6]. However, the lower swelling ratio (0.35 g/g) and higher density (3.60 g/cm^3^, Figure 8) compared to Gu/DexS/RC suggest a more compact matrix, likely delaying active release. The mechanical reinforcement observed (Young’s modulus ~ 1.70 MPa, Table 7 and Figure 9) correlates with the rougher, aggregated morphology seen in the SEM images (Figure 10C), which may limit matrix flexibility but improve sustained activity.

Interestingly, Gu/DexS/Li/RC showed slightly reduced inhibition (48.70%) compared to RC or Li alone. This suggests potential competitive interactions between polyphenols and lignin at the molecular level, possibly hindering optimal release or surface availability. These findings align with the observed mechanical stiffening (Young’s modulus ~ 2.83 MPa) and increased matrix density (2.33 g/cm^3^), both of which may restrict the diffusion of actives.

The esterification of lignin with aspartic acid (LiAs) aimed to enhance compatibility with the hydrophilic matrix and introduce additional bioactivity. Although aspartic acid is known to reduce oxidative stress [36], the Gu/DexS/LiAs/RC formulation presented a modest inhibition effect (~46%), slightly lower than Gu/DexS/Li. This may be due to the denser, phase-separated morphology (Figure 10F), which can entrap active compounds and reduce immediate bioavailability.

Drug release kinetics further explain this observation. According to the Makoid–Banakar model (Table 8), the rate constant (k) decreased significantly from Gu/DexS/RC (5.84) to Gu/DexS/Li/RC (4.38) and Gu/DexS/LiAs/RC (3.24), indicating delayed RC extract release. Additionally, the exponential decay factor (a) and release exponent (n) dropped across the same series, reflecting a shift from rapid to more diffusion-controlled release (n values: 0.635 → 0.451 → 0.573). These trends correlate with the reduced swelling ratios and increasing matrix rigidity, confirming that the delayed but sustained release profile in lignin-containing systems contributes to prolonged, though less immediate, anti-inflammatory action.

Overall, anti-inflammatory efficacy across the materials is determined by a fine balance between structural compactness, filler chemistry, mechanical behavior, and drug release dynamics. Gu/DexS/RC offers the fastest and most potent activity due to rapid swelling and RC diffusion, while Gu/DexS/Li and Gu/DexS/LiAs provide sustained effects through reinforced matrices and delayed release, making them valuable for prolonged skin therapies.

### 3.12. In Vitro Biocompatibility of Materials

**Figure 13:**
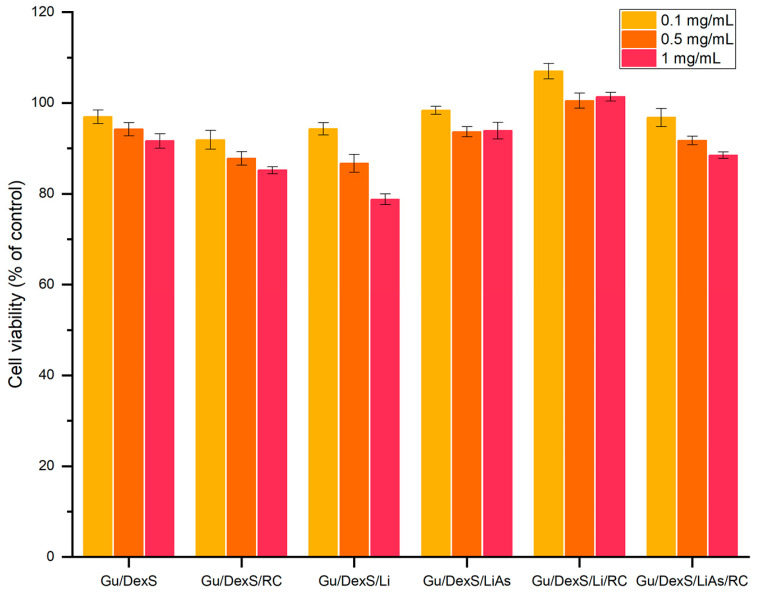
The biocompatibility of the obtained materials on normal fibroblasts (24 h).

The in vitro biocompatibility of the Gu/DexS-based materials was assessed using the MTS assays on human gingival fibroblasts. All tested formulations exhibited high cell viability across concentrations of 0.1, 0.5, and 1 mg/mL, with values consistently above 80% (Figure 13), confirming their cytocompatibility and potential for dermatological use.

The reference matrix, Gu/DexS, showed excellent viability (92–97%), attributed to the known non-toxic, hydrophilic nature of polysaccharides, which promote favorable cell adhesion and proliferation [46]. The SEM images (Figure 10A) revealed a smooth, uniform surface, while mechanical testing (Figure 9) indicated moderate stiffness (Young’s modulus ~ 1.42 MPa), supporting its ability to maintain a soft, compliant interface conducive to cell growth.

The addition of *Rosa canina* extract slightly reduced viability at higher concentrations (85% at 1 mg/mL), likely due to the polyphenolic content exerting mild pro-oxidative or dose-dependent effects [47]. Despite this, Gu/DexS/RC maintained robust cellular response while achieving the highest anti-inflammatory effect (61.58% inhibition of albumin denaturation, Figure 12), indicating a balanced bioactivity–biocompatibility profile.

Materials containing unmodified lignin (Gu/DexS/Li) exhibited lower cell viability at 1 mg/mL (~79%), likely due to the hydrophobic, aromatic character of lignin and its rougher surface morphology (Figure 10C), which may hinder cell adhesion and nutrient exchange. Mechanical reinforcement (Young’s modulus ~ 1.70 MPa) further reflects this rigidity, possibly compromising cellular spreading.

By contrast, the esterification of lignin with aspartic acid (LiAs) significantly improved biocompatibility. Gu/DexS/LiAs showed cell viability of 94–98%, a direct result of increased polarity and hydrophilicity conferred by amino and carboxylic groups in LiAs [48,49]. Though the SEM images (Figure 10E) displayed visible morphological heterogeneity, the improved chemical affinity with the Gu/DexS matrix likely reduced interfacial tension, supporting better cellular interaction. The associated mechanical properties (Young’s modulus ~ 2.2 MPa) reflect a denser but more functionally integrated matrix.

The most favorable biological response was observed for Gu/DexS/LiAs/RC, which combined high cell viability (88–100%) with strong anti-inflammatory activity (46.23%) and enhanced mechanical performance (Young’s modulus ~ 3.74 MPa) (Figure 9). The SEM micrographs (Figure 10F) revealed heterogeneous morphology with large aggregates, suggesting strong intermolecular interactions. Despite this structural density, drug release kinetics (Table 8) remained controlled (*k* ≈ 3.24; *n* ≈ 0.57, Makoid–Banakar model), allowing the sustained delivery of RC bioactive compounds and reducing cytotoxic peaks.

The pseudo-second-order swelling behavior observed for all materials (Figure 8, Table 6) further supports their favorable hydration kinetics, gradually increasing water uptake without sudden volumetric expansion, thus preserving the integrity of the cell–material interface over time [23].

In summary, the biocompatibility of the tested materials is closely tied to their structural and functional features. The integration of LiAs and RC extract within the Gu/DexS matrix creates a balance between mechanical integrity, sustained bioactive release, and cellular tolerance. The Gu/DexS/LiAs/RC formulation emerges as the most promising candidate for skin-contact biomaterials, combining reinforced structure, antioxidant and anti-inflammatory bioactivity, and excellent cytocompatibility.

## 4. Conclusions

This research introduces an innovative platform of multifunctional biomaterials based on natural polysaccharides, guar gum and dextran sulfate, engineered through the incorporation of softwood lignin (native and esterified) and a polyphenol-rich *Rosa canina* extract. The study addressed a key challenge in the design of bio-based skin-contact materials: achieving a balance between biocompatibility, structural integrity, controlled bioactive delivery, and anti-inflammatory function, using sustainable and accessible resources.

A central novelty of the study lies in the enzymatic chemical modification of lignin with aspartic acid, which enhanced its polarity and compatibility with hydrophilic matrices. This approach enabled a more homogeneous dispersion of the filler, improving both mechanical cohesion and biological integration. Combined with *Rosa canina* extract, known for its antioxidant and therapeutic potential, the developed materials demonstrate synergistic effects between the plant-derived components, exceeding the performance of individual fillers.

Across all formulations, the influence of filler type and interaction was evident in the materials’ structural, mechanical, and functional behavior. The dual reinforcement strategy, using both esterified lignin and bioactive extract, led to denser and more resilient matrices, with improved tensile performance, reduced swelling capacity, and a sustained release profile that extended the functional window of the incorporated substances. These findings highlight the versatility of the Gu/DexS matrix as a customizable platform for biomedical and cosmetic uses.

Importantly, all systems maintained high cytocompatibility with human fibroblasts, validating their potential for skin-contact applications. The improved cell viability in the presence of esterified lignin underlines the benefits of strategic chemical functionalization for reducing cytotoxicity while maintaining mechanical strength. Additionally, the in vitro anti-inflammatory activity of the RC-containing systems supports their relevance for topical therapeutic use, particularly in products aimed at calming irritation, inflammation, or oxidative stress.

In summary, this study demonstrates that, by integrating naturally derived polysaccharides, functionalized lignin, and plant extracts, it is possible to obtain advanced materials with a tuned balance of mechanical robustness, functional delivery, and biological safety. Among all tested variants, the Gu/DexS/LiAs/RC formulation stood out as a lead candidate, offering enhanced performance without compromising eco-compatibility. These findings contribute to the growing field of sustainable bio-based materials for dermal systems that are both effective and environmentally responsible.

## Figures and Tables

**Table 1:** Materials composition.

Material	Mass Ratio *
GU/DexS	2:1
GU/DexS/Ma	2:1:0.8
GU/DexS/Li	2:1:0.8
GU/DexS/Li/Ma	2:1:0.8:0.8
GU/DexS/LiAs	2:1:0.8
GU/DexS/LiAs/Ma	2:1:0.8:0.8

* Ratios refer to the dry mass of neat components.

**Table 2:** Chemical composition of Li.

Element	C	O	S
Relative concentration (%)	77.50	21.64	0.85

**Table 3:** Proportions of oxygen atom in different functional groups.

Assignment	O1	O2	O3
Binding energy (eV)	531.9	533.3	534.3
Relative concentration (%)	10.01	76.98	13.01

**Table 4:** Proportions of carbon atom in different functional groups.

Assignment	C1	C2	C3	C4
Binding energy (eV)	285.0	286.5	287.5	289.0
Relative concentration (%)	57.94	35.24	5.33	1.49

**Table 5:** Chemical composition of LiAs.

Element	BE [eV]	FWHM [eV]	RSF	Atomic Conc. [%]	Error [%]	Mass Conc. [%]	Error [%]
O 1s	531.0	3.92	0.78	22.98	0.19	28.01	0.22
N 1s	398.0	4.86	0.48	3.83	0.27	4.09	0.28
C 1s	286.0	6.21	0.28	72.57	0.27	66.40	0.30
S 2p	165.0	8.52	0.67	0.62	0.07	1.51	0.18

**Table 6:** The fitting data of the obtained materials with different swelling models.

Material	Correlation Coefficient (R^2^)	
	First Order	Pseudo-Second Order
Gu/DexS	0.945	0.991
Gu/DexS/RC	0.957	0.994
Gu/DexS/Li	0.987	0.996
Gu/DexS/Li/RC	0.983	0.995
Gu/DexS/LiAs	0.923	0.998
Gu/DexS/LiAs/RC	0.841	0.997

**Table 7:** Elongation at break of the obtained materials.

Material	Elongation at Break, %
Gu/DexS	348.8 ± 72.9
Gu/DexS/RC	251.9 ± 98.0
Gu/DexS/Li	285.1 ± 91.3
Gu/DexS/Li/RC	264.4 ± 30.2
Gu/DexS/LiAs	203.4 ± 10.9
Gu/DexS/LiAs/RC	237.8 ± 31.1

**Table 8:** The kinetic parameters of mathematical models applied to the experimental data concerning the release of RC extract from the tested materials.

	Gu/DexS/RC	Gu/DexS/Li/RC	Gu/DexS/LiAs/RC
Weibull			
*A*	95.84953 ± 1.3206	76.02097 ± 36.14333	76.34455 ± 12.3027
*x_c_*	−3.260 × 10^−9^ ± 0.09504	−2.37675 × 10^−31^ ± 0.69839 × 10^−16^	−4.73495 × 10^−5^ ± 0.02678
*d*	0.89583 ± 0.0476	0.49052 ± 0.09834	0.65189 ± 0.07492
*k*	0.02077 ± 8.70968 × 10^−4^	0.00299 ± 0.00467	0.00635 ± 0.00279
*red-χ* ^2^	3.77609	2.14893	2.48877
R^2^	0.99464	0.98414	0.98997
Korsmeyer–Peppas			
*k*	17.62458 ± 2.57874	5.93382 ± 0.53193	5.39583 ± 0.51909
*n*	0.31692 ± 0.02914	0.36534 ± 0.01774	0.42997 ± 0.0189
*red-χ* ^2^	32.73172	2.04145	3.26334
R^2^	0.95358	0.98493	0.98685
Higuchi			
*k*	6.9897 ± 0.20888	3.00387 ± 0.05908	3.78486 ± 0.04931
*red-χ* ^2^	100.12979	8.0103	5.58048
R^2^	0.85799	0.94088	0.97751
Makoid–Banakar			
*k*	5.83796 ± 0.91091	4.37995 ± 0.87828	3.23549 ± 0.63449
*a*	0.00293 ± 3.55382 × 10^−4^	7.59458 × 10^−4^ ± 4.568 × 10^−4^	0.00123 ± 4.23933 × 10^−4^
*n*	0.63527 ± 0.04174	0.45136 ± 0.05382	0.57316 ± 0.05186
*red-χ* ^2^	5.65783	1.8442	2.19185
R^2^	0.99198	0.98639	0.99117
Gompertz			
*a*	92.16327 ± 1.49361	42.79827 ± 1.51389	55.5579 ± 1.88074
*b*	2.17391 ± 0.25208	1.68256 ± 0.20484	1.87366 ± 0.20066
*c*	0.03302 ± 0.00332	0.01912 ± 0.00293	0.01815 ± 0.0024
*red-χ* ^2^	18.23845	7.60159	9.69633
R^2^	0.97413	0.9439	0.96092
Hixson–Crowell			
*k*	0.02188 ± 0.00133	0.00416 ± 2.85978 × 10^−4^	0.00566 ± 3.294 × 10^−4^
*red-χ* ^2^	45.64247	64.21544	63.06091
R^2^	0.93527	0.52605	0.74585

## Data Availability

The data are contained within the article.
